# Return to Work After a Cardiovascular Event: The Central Role of Cardiac Rehabilitation

**DOI:** 10.3390/jcm15052019

**Published:** 2026-03-06

**Authors:** Mario Pacileo, Francesco Giallauria, Gianluigi Cuomo, Giuseppe Vallefuoco, Alfredo Mauriello, Vincenzo Russo, Antonello D’Andrea

**Affiliations:** 1 Cardiology and Intensive Care Unit, Department of Cardiology, “Umberto I” Hospital, 84014 Nocera Inferiore, Italy; m.pacileo@aslsalerno.it (M.P.); antonellodandrea@libero.it (A.D.); 2 Department of Translational Medical Sciences, Federico II University of Naples, 80131 Naples, Italy; 3 Department of General Medicine, “Ospedale del Mare”, ASL Napoli 1Centro, 80147 Naples, Italy; gianluigi.cuomo@aslnapoli1centro.it; 4 Cardiology Unit, University of Campania “Luigi Vanvitelli”, 80138 Naples, Italy; giuseppevallefuoco46@gmail.com; 5 Cardiology Unit, Institute National Cancer, IRCCS, Foundation “G. Pascale”, 80131 Naples, Italy; alfredo.mauriello93@libero.it; 6 Cardiology Unit, Monaldi Hospital, Department of Medical and Translational Sciences, University of Campania “Luigi Vanvitelli”, 80138 Naples, Italy; vincenzo.russo@unicampania.it

**Keywords:** return to work, cardiac rehabilitation, acute coronary syndrome, heart failure, depression, self-efficacy, METs, CPET, telerehabilitation, occupational medicine, quality of life

## Abstract

**Background:** Return to work (RTW) after acute coronary syndrome (ACS) or acute heart failure (HF) is a pivotal outcome reflecting functional recovery and quality of life (QoL). While survival after cardiac events has improved through reperfusion and guideline-directed pharmacotherapy, sustainable RTW depends on an integrated set of clinical, psychological, social, and occupational determinants. **Objective:** This study aimed to synthesize and expand the evidence on predictors of RTW, delineate practical workload-matching rules using METs and CPET, and position multidisciplinary cardiac rehabilitation (CR) as the bridge from clinical recovery to durable vocational reintegration. **Key findings:** Beyond left ventricular ejection fraction (LVEF), depression, anxiety, illness perceptions, and RTW self-efficacy are robust predictors of vocational outcomes. CPET-guided exercise prescriptions and MET-based job matching ensure adequate metabolic reserve; sustained task demand should remain at ≤35–40% of maximal capacity, with peak capacity ≥2× average job demand. CR (Class IA in the 2023 ESC ACS Guidelines) improves exercise tolerance, medication adherence, psychosocial well-being, and deployment of vocational support, including stepwise reintegration plans and ergonomic adaptations. Telerehabilitation extends monitoring and counseling into the workplace and maintains adherence after RTW. **Conclusions:** Comprehensive CR that integrates exercise training, psychosocial counseling, lifestyle modification, and vocational interventions offers the most effective pathway to stable RTW, improved QoL, and reduced socio-economic burden. Early identification of vulnerable subgroups and personalized, digitally supported follow-up are essential for long-term job retention.

## 1. Introduction

Return to work (RTW) after a cardiovascular event such as acute coronary syndrome (ACS) or acute heart failure (HF) encapsulates physical capacity, psychological readiness, and socio-occupational functioning. Despite increasing post-event survival, stable employment depends on coordinated clinical, functional, psychosocial, and occupational factors [1,2].

CR integrates exercise training, risk factor modification, medication optimization, psychosocial care, and vocational support—linking hemodynamic recovery to occupational performance over time [2,3,4]. In this narrative review, observational cohorts, registries, meta-analyses, CR trials, and guideline statements were analyzed focusing on biopsychosocial predictors, CPET/METs assessment, CR components, vocational support, telerehabilitation, and disparities. We foreground employment trajectories [1], determinants of sustained RTW [2,5,6,7], prognostic imaging [8,9,10,11,12,13], and vocational reintegration within CR [2,3,14,15,16,17], cross-validating with ESC/EAPC guidelines [4,18,19].

Predictors, workload-matching rules, implementation strategies, telerehabilitation, disparities, and a practical algorithm to translate capacity gains into safe workplace exposure were also discussed.

## 2. Epidemiology and Socio-Economic Impact

Ischemic heart disease remains a principal cause of mortality worldwide, and a substantial proportion of myocardial infarctions (MI) occur among individuals in the active workforce, amplifying both clinical and socioeconomic repercussions [5,20,21]. Beyond the immediate burden of acute care, MI triggers significant indirect costs driven by prolonged sick leave, reduced productivity, and premature exit from employment. In national analyses, for example, first-year post-MI productivity losses in Portugal exceed €10 million, illustrating the tangible economic impact of work disruption following cardiac events [22]. Large registry data offer further insight into the dynamics of return to work (RTW). Approximately 91% of working-age MI survivors resume employment within one year, yet nearly 24% disengage from the workforce during the subsequent year, despite initial reintegration [1]. This pattern reflects the persistence of post-MI challenges that are not fully captured by standard clinical recovery metrics—namely fluctuating physical endurance, lingering symptoms, maladaptive illness perceptions, and workplace-related anxiety. These findings underscore the need for a coordinated, multidisciplinary framework that extends beyond routine cardiology follow-up [2,6]. Integrating cardiologists, rehabilitation specialists, psychologists, and occupational physicians facilitates early identification of functional or psychosocial vulnerabilities and enables proactive modification of rehabilitation and vocational plans. Such an approach supports sustainable work participation, reduces the risk of secondary job loss, and mitigates long-term societal costs. By embedding vocational considerations into cardiac rehabilitation and follow-up pathways, healthcare systems can more effectively address the complex interplay between clinical recovery, functional capacity, and workplace reintegration that characterizes post-MI trajectories.

## 3. Predictors of Returning to Work

### 3.1. Psychological Factors

Psychological variables play a decisive role in determining whether cardiac patients successfully resume employment after an acute event. Among these, depression and anxiety are consistently identified as the strongest negative predictors of RTW [7,23]. Early depressive symptoms—often detectable within the first one to two weeks after the event—can impair motivation, reduce confidence in physical recovery, and heighten perceptions of vulnerability, ultimately delaying or preventing re-entry into the workforce. Even mild affective symptoms may accumulate to create a substantial psychological barrier, demonstrating a dose–response relationship in which greater severity corresponds to poorer vocational outcomes [7,23]. A second key factor involves illness perceptions, particularly beliefs about fragility, permanent damage, or elevated risk of recurrence. These cognitive appraisals can persist even when objective physical capacity has normalized, causing patients to underestimate their functional abilities. Such maladaptive perceptions frequently lead to avoidance of activity, restriction of effort, and reluctance to engage in job tasks perceived as strenuous or stressful [2,6,24]. Work-related cognition also contributes meaningfully. Effort–Reward Imbalance, in which patients perceive high demands but insufficient support or recognition, undermines motivation and increases psychological strain during recovery [25]. This imbalance can interact with residual somatic symptoms, reinforcing doubts about workplace sustainability. Conversely, RTW self-efficacy—the belief in one’s ability to resume and maintain occupational roles—emerges as a strong positive predictor. Individuals with higher self-efficacy show earlier, more stable reintegration, greater adherence to rehabilitation activities, and more adaptive coping behaviors [15,24,25,26,27].

Recent meta-analytic evidence indicates that psychologically enhanced CR shows additional benefits in quality of life and functional outcomes compared with exercise-only CR, supporting the value of structured psychosocial modules within modern CR programs [28]. In a recent clinical trial, integration of psychosocial support alongside aerobic training produced significantly greater improvements in psychological well-being and health-related quality of life than conventional CR alone [29]. Targeted counseling that strengthens mastery, enhances problem-solving skills, and reframes catastrophic thoughts can substantially improve readiness for RTW. Integrating these interventions within cardiac rehabilitation ensures that psychological recovery progresses in parallel with physical recovery, supporting a safer and more durable return to employment.

### 3.2. Clinical and Echocardiographic Considerations

Left ventricular systolic function is a cornerstone determinant of vocational prognosis after myocardial infarction (MI) and acute coronary syndromes. Reduced left ventricular ejection fraction (LVEF) is consistently associated with higher mortality risk and greater long-term disability, which translate into a higher likelihood of work limitation and early retirement, particularly in jobs requiring sustained physical effort or safety-critical performance [8]. Functional impairment in this setting often reflects limited cardiopulmonary reserve, exertional dyspnea, fatigue, and lower tolerance to workload peaks, all of which can compromise sustainable return to work (RTW).

Beyond conventional LVEF, global longitudinal strain (GLS) provides a more sensitive marker of myocardial function. GLS can identify subclinical ventricular dysfunction even when LVEF is preserved, and it has prognostic value in anticipating adverse remodeling following MI, thereby supporting earlier risk stratification for delayed recovery and potential work restrictions [9,10,11]. In practical terms, GLS can help identify patients who may appear clinically stable yet remain at risk for functional decline over the ensuing months, a period that often overlaps with attempts to resume employment. Emerging imaging approaches evaluating left ventricular vortex flow patterns add a complementary layer of pathophysiological insight. Abnormal vortex formation may reflect impaired energetic efficiency of ventricular filling and ejection and may also relate to conditions that increase thrombotic propensity, offering additional clues about residual vulnerability after MI [12,13]. While not routinely used for occupational decisions, such measures can refine risk profiling in selected cases. Acute clinical severity indices remain highly relevant. A higher Killip class, reflecting greater heart failure severity during the acute phase, is linked to reduced functional capacity and more complicated recovery trajectories that can delay or prevent RTW [30]. Similarly, multimorbidity (e.g., diabetes, chronic kidney disease, or pulmonary disease) amplifies symptom burden and limits physiologic reserve, further diminishing work capacity and tolerance to job demands [20].

Beyond systolic indices, arrhythmic burden and coronary disease complexity also influence vocational prognosis. Evidence from the ESC Guidelines for ventricular arrhythmias highlights that recurrent ventricular arrhythmias, atrial fibrillation with poor rate control, or recent ICD therapies are associated with increased morbidity, safety concerns, and psychological distress, all of which may delay RTW [31]. Similarly, greater anatomical complexity of coronary artery disease—particularly multivessel or residual ischemia—has been associated with higher recurrence risk and functional limitation [32], potentially affecting confidence and work sustainability. Recurrent MI represents a strong negative predictor of durable employment, often compounding deconditioning and illness perceptions [33]. Age acts as a modifier: older patients may face reduced physiologic reserve, whereas younger individuals, despite preserved capacity, appear vulnerable to long-term occupational detachment due to psychosocial and labor-market factors [34].

Finally, patients with MI complicated by cardiogenic shock represent a high-risk subgroup with markedly reduced RTW probability. Prolonged hospitalization, deconditioning, and potential anoxic brain injury can compound physical and cognitive limitations, often necessitating prolonged rehabilitation and individualized vocational planning [35].

### 3.3. Functional Capacity and MET-Based Job Matching

Functional capacity assessment is a cornerstone of safe vocational reintegration following myocardial infarction (MI) or acute coronary syndromes. It provides an objective physiological framework for determining whether a patient possesses sufficient cardiopulmonary reserve to meet the metabolic demands of their specific occupation. Central to this process is the metabolic equivalent of task, where 1 MET corresponds to approximately 3.5 mL/kg/min of oxygen consumption [25]. This standardized unit allows clinicians to translate fitness measurements into meaningful thresholds for job-task evaluation.

Cardiopulmonary exercise testing (CPET) is the gold standard for quantifying functional capacity, providing precise measurements of VO_2_peak, ventilatory thresholds, and hemodynamic responses necessary for individualized exercise prescriptions and return-to-work (RTW) decision-making. By integrating CPET findings with known MET demands of occupational tasks, clinicians can determine the degree of physiological reserve required for safe and sustained work performance.

Evidence-based workload-matching principles further refine this process. Exercise physiology principles and occupational medicine consensus recommendations suggest that sustained occupational workload should not exceed 35–40% of maximal capacity over a typical 6–8 h shift [36,37]. This ensures adequate metabolic buffer to prevent excessive fatigue, symptom exacerbation, or cardiovascular instability during prolonged work periods. Additionally, the peak functional capacity should be at least twice the average job MET requirement, providing sufficient reserve for unplanned effort spikes, task variability, or environmental stressors [38].

A practical illustration is a job requiring 4 METs—such as brisk walking, light industrial activity, or certain service-sector roles—which would necessitate a minimum peak capacity of ≥8 METs, corresponding to approximately 28 mL/kg/min VO_2_peak [38] (Table 1). This quantitative matching facilitates transparent communication between clinicians, occupational health specialists, and employers, helping structure graded RTW plans, identify necessary duty modifications, and prevent premature or unsafe occupational exposure.

Ultimately, integrating MET-based thresholds with CPET-derived data enables a systematic, reproducible, and patient-specific approach to RTW planning, reducing the risk of recurrent symptoms, overexertion, or work detachment during the critical months following cardiac events.

## 4. The Role of Cardiac Rehabilitation

Cardiac Rehabilitation (CR) is a Class I, Level A recommendation in the 2023 ESC Acute Coronary Syndrome (ACS) guidelines, reflecting the highest level of evidence and consensus that CR should be offered as a core component of post-event care [2,4,18]. CR is delivered through a multidisciplinary team that typically includes cardiologists, rehabilitation physicians, physiotherapists/exercise physiologists, specialist nurses, dietitians, psychologists, social workers, and—when return to work (RTW) is a goal—occupational physicians and ergonomists who translate functional capacity into safe job demands and workplace accommodations [2,3,14,15,16,17]. The “exercise core” of CR remains central because improvements in cardiorespiratory fitness represent a potent, measurable pathway to better outcomes; nevertheless, the strongest programs treat exercise as one element of a broader biopsychosocial model, recognizing that sustained recovery depends on adherence, emotional adjustment, health beliefs, workplace context, and social support. Importantly, robust evidence indicates that CR participation is associated with meaningful clinical benefit: a large contemporary meta-analysis of 85 randomized trials (n ≈ 23,430) of exercise-based CR for coronary heart disease found significant reductions in cardiovascular mortality (RR 0.74; 95% CI 0.64–0.86; NNT ≈ 37) and hospitalizations (RR 0.77; 95% CI 0.67–0.89; NNT ≈ 37), as well as fewer recurrent myocardial infarctions (RR 0.82; 95% CI 0.70–0.96), alongside evidence of improved health-related quality of life (HRQoL) and cost-effectiveness. These outcome statistics underscore that CR delivers benefits not only in functional capacity and patient-reported health, but also in “hard” endpoints relevant to health systems and employers, such as cardiovascular death and rehospitalization. Complementing trial-level evidence, real-world cohort data also show substantial associations between completing CR and survival: in a large retrospective cohort of 11,196 post-ACS referrals (mean follow-up 4.2 years), CR completion was associated with lower all-cause mortality (adjusted HR 0.67) and lower cardiovascular mortality (adjusted HR 0.57), together with improvements in fitness, lipid control, body composition, smoking rates, and psychological distress. While observational estimates are vulnerable to selection effects, these findings are consistent with the mechanistic concept that CR improves prognosis through risk factor modification, fitness gains, and behavioral adherence maintained over time. Within this multidimensional framework, psychological care is not an optional add-on but a clinical necessity, because depression, anxiety, illness perceptions, and low self-efficacy strongly shape recovery trajectories, adherence, and vocational outcomes after MI/ACS. A major challenge is that post-event psychological distress is common yet frequently under-recognized: systematic review evidence shows that depression predicts delayed or failed RTW in substantial proportion of studies, with a trend toward a dose–response relationship whereby greater depression severity is associated with poorer RTW outcomes at 6–12 months [4,19,39]. For this reason, contemporary CR programs increasingly embed routine screening using validated instruments such as the Hospital Anxiety and Depression Scale (HADS) or Patient Health Questionnaire (PHQ), enabling early identification of patients who may otherwise appear “cardiologically stable” but remain functionally limited by fear, fatigue, low mood, sleep disturbance, catastrophizing, or avoidance behaviors. Recent studies indicate that psychological interventions in cardiac populations can yield clinically relevant improvements in mental health outcomes: a Cochrane systematic review and meta-analysis of 21 RCTs (n ≈ 2591) found that psychological interventions reduced depression (SMD −0.36) and anxiety (SMD −0.57) and improved mental HRQoL (SMD 0.63) at 6–12 months of follow-up, with targeted anxiety-focused interventions showing greater effects than non-targeted approaches. Beyond meta-analytic averages, pragmatic CR-integrated trials also demonstrate that structured psychological modules can be delivered effectively within routine services: in the PATHWAY randomized controlled trial of group metacognitive therapy added to CR (n = 332), the combined intervention produced significant improvements in total HADS score at 4 months (between-group difference −3.24; standardized effect size ~0.52) that persisted at 12 months, without treatment-related adverse events—illustrating that scalable, theory-based psychological treatments can meaningfully reduce distress during the vulnerable post-event period. These psychological gains matter not only for well-being but also for “behavioral endpoints” that influence prognosis and work sustainability: mood and anxiety symptoms affect medication adherence, participation in physical training, sleep quality, and confidence in resuming daily tasks, including employment. Indeed, evidence from employed post-MI cohorts suggests that continuing CR is associated with better psychosocial functioning after RTW. In the study of middle-aged post-AMI workers comparing phase II CR participants with non-participants, those who attended CR showed better scores for psychological distress subscales and higher HRQoL at six months, whereas discontinuation of CR was associated with chronic psychosocial stress after RTW; notably, this program included counseling components where therapists monitored symptoms, fatigue, and concerns related to working life, indicating how structured follow-up can address day-to-day barriers that emerge once patients are back in their occupational environment. In parallel, vocational support within CR provides the operational “bridge” from clinical recovery to safe job performance. Effective vocational CR involves a detailed job-demand history (physical load, shift work, heat/cold exposure, psychosocial stressors), functional capacity evaluation (often using CPET and MET-based frameworks), and coordinated communication—respecting privacy—between the clinical team, occupational health services, and employers to enable phased return, graded duty escalation, and ergonomic adaptation. The best-performing CR models treat psychological screening and intervention as routine, not exceptional, using standardized tools (HADS/PHQ), targeted therapies when distress is clinically significant, and continuous coaching to promote adherence, confidence, and self-efficacy. When implemented in this comprehensive manner, CR can reduce cardiovascular mortality and hospitalization risk in coronary populations, improve quality of life, lower psychological distress, and support a safer, faster, and more sustainable return to work—thereby delivering benefits that are simultaneously medical, psychosocial, and socioeconomic.

## 5. Measuring Occupational Outcomes: Work Performance Scale (WPS)

The Work Performance Scale (WPS), a core component of the Functional Status Questionnaire (FSQ), provides a structured and validated method for quantifying post-event occupational functioning in cardiac patients [40]. Scored from 1 to 4, with higher scores reflecting better work performance, the WPS captures domains such as reliability, task completion, concentration, and perceived ability to sustain productivity across a typical workday. This makes it particularly suited for evaluating recovery in individuals returning to the workplace after acute coronary syndrome (ACS), myocardial infarction (MI), or cardiac surgery, where subtle deficits in endurance, cognitive efficiency, or emotional regulation can substantially influence work sustainability even when traditional clinical parameters appear stable. Evidence consistently demonstrates that participation in cardiac rehabilitation (CR) is associated with higher WPS scores, fewer sick leave days, and reduced levels of anxiety and depression [14,15,16,17,41]. These gains are especially prominent among white-collar workers, whose job roles depend heavily on cognitive focus, decision-making, and psychosocial resilience—domains positively impacted by the comprehensive biopsychosocial model of CR. Studies also show that patients engaged in CR report stronger confidence in their ability to handle work demands, better symptom self-management, and a lower likelihood of early work detachment or recurrent absence. Routine integration of WPS into the RTW process strengthens clinical oversight and vocational planning. Weekly WPS assessments during a trial RTW phase allow clinicians and occupational physicians to identify early warning signs such as excessive fatigue, difficulty concentrating, impaired stress tolerance, or mismatch between functional capacity and job demands. Transitioning to monthly WPS monitoring thereafter supports ongoing adjustment of work hours, task intensity, ergonomic accommodations, and psychological support strategies. This proactive approach helps maintain employment stability while preventing relapse, burnout, or unnecessary long-term disability. By providing an objective and sensitive measure of real-world functioning, the WPS serves not only as an outcome indicator but also as a dynamic clinical decision tool, ensuring that return to work is safe, progressive, and sustainable.

## 6. From the Acute Phase to Telerehabilitation

Telerehabilitation and hybrid CR models—combining center-based sessions with remote monitoring and coaching—can extend supervision into the workplace and have demonstrated outcomes comparable to traditional programs for functional capacity and secondary-prevention targets while improving accessibility and adherence. Telerehabilitation integrates remote monitoring, coaching, and education; trials show comparability to center-based CR for functional gains and risk factor targets [42,43,44,45].

Recent guideline-level standards on telerehabilitation emphasize structured remote CR as a quality indicator and feasible alternative to centre-based programs, supported by controlled cohorts evaluating functional outcomes and adherence [46]. Implementation should follow ESC e-Cardiology guidance (privacy, usability, interoperability) [43]. Hybrid models offer frequent touchpoints without work disruption. In hybrid and telerehabilitation models, remote monitoring enhances safety and clinical effectiveness by enabling continuous oversight of symptoms, heart rate, rhythm, exercise adherence, and psychological status, thereby sustaining engagement and achieving functional and secondary-prevention outcomes comparable to traditional center-based CR while improving accessibility and long-term adherence.

## 7. Gender Disparities and Socio-Occupational Barriers

Gender and occupational context exert a substantial influence on return-to-work (RTW) trajectories following myocardial infarction or acute coronary syndromes. Women consistently exhibit lower RTW rates, a pattern attributed to a complex interplay of factors including a higher burden of comorbid conditions, greater prevalence of post-event depression, and persistent sociocultural expectations that shape caregiving roles and limit prioritization of personal recovery [47,48,49]. These factors may amplify perceived or actual functional limitations and contribute to delayed vocational reintegration even when clinical recovery is satisfactory.

Occupational class further modulates RTW outcomes. Individuals in blue-collar roles face disproportionately greater challenges due to the higher physical demands of their jobs, which may exceed post-MI capacity thresholds or require prolonged training and ergonomic adjustments before safe resumption is feasible [39]. Conversely, the self-employed often return to work more rapidly because of financial pressure and limited access to paid sick leave; however, this early return may be accompanied by greater psychological and economic stress, potentially reducing long-term sustainability of employment [50,51].

In contrast, white-collar workers tend to face fewer physical barriers but may experience significant psychosocial strain, including cognitive fatigue, anxiety, and high responsibility loads. These pressures can adversely affect the Work Performance Scale (WPS) and contribute to reduced job satisfaction or performance impairments during early reintegration phases [16,41].

Age-related patterns also warrant attention. Younger adults, particularly those aged 30–39 years, demonstrate a paradoxical trend: although medically fit, they are at heightened risk for long-term detachment from the workforce following initial RTW. This may reflect instability in early career trajectories, financial insecurity, or heightened psychosocial vulnerability in younger populations [1].

Collectively, these disparities highlight the need for individualized, equity-oriented RTW strategies that consider gender, occupation, and age-specific challenges when designing cardiac rehabilitation and vocational planning pathways.

## 8. Pathophysiological and Behavioral Linkages Between Work Stress and Cardiac Risk

The relationship between occupational stress and cardiovascular vulnerability is grounded in well-established pathophysiological pathways. High-strain work environments characterized by high demand and low control provoke sustained activation of the sympathoadrenal system, leading to elevated catecholamine levels, increased heart rate, higher blood pressure, and heightened myocardial oxygen demand—conditions that cumulatively accelerate atherosclerotic progression and destabilize plaque morphology [2]. Similarly, effort–reward imbalance, where occupational effort is chronically disproportionate to perceived reward, reinforces maladaptive stress responses and contributes to neuroendocrine dysregulation, endothelial dysfunction, and systemic inflammation [4]. Chronic exposure to such imbalances has been associated with impaired nitric-oxide-mediated vasodilatation and increased pro-thrombotic tendency, which further elevates cardiovascular risk.

Work schedule characteristics also exert significant influence. Shift work, particularly involving circadian disruption, impairs metabolic regulation, increases sympathetic tone, and alters cortisol secretion patterns, thereby amplifying cardiovascular strain and predisposing individuals to both acute and chronic ischemic events [18]. These physiological perturbations decrease resilience to workload fluctuations and may undermine recovery trajectories following cardiac rehabilitation.

Integrated behavioral and organizational interventions can mitigate these risks. Psychophysiological strategies, including breathing retraining, mindfulness-based stress reduction, and cognitive reframing, help attenuate sympathetic arousal, reduce perceived stress, and support adaptive coping mechanisms [25]. At the organizational level, workplace policies promoting micro-breaks, adequate thermal and environmental control, and task rotation reduce cumulative allostatic load by moderating physical and cognitive strain across the workday. Such adjustments not only support cardiovascular safety but also sustain performance, engagement, and long-term employability following cardiac events.

Together, these mechanisms underscore the necessity of incorporating both behavioral and occupational interventions into post-event care pathways, ensuring that return-to-work decisions account for the physiological consequences of work-related stress exposures.

## 9. Medication Optimization and Adherence in the Context of RTW

Optimal pharmacologic management is a foundational component of post-event recovery and plays a central role in determining whether patients can safely and sustainably return to work. Adherence to guideline-directed medical therapies—including antiplatelet agents, beta-blockers, renin–angiotensin–aldosterone system inhibitors, statins, and, when appropriate, SGLT2 inhibitors or mineralocorticoid receptor antagonists—has been shown to stabilize symptoms, reduce recurrent ischemic risk, and improve overall functional tolerance during daily activities and occupational tasks [4,18]. Medication adherence directly influences exercise capacity, autonomic stability, and symptom perception, making it a critical determinant of readiness for work resumption.

CR provides an ideal structure for comprehensive medication review and optimization. Through multidisciplinary oversight, CR teams assess pharmacologic regimens for appropriateness, titration needs, contraindications, and potential interactions affecting exercise performance or occupational safety. Particular attention is given to side-effect profiles—such as fatigue, orthostatic dizziness, sleep disturbance, or musculoskeletal symptoms—which may hinder participation in rehabilitation, impair job performance, or discourage adherence if unaddressed.

CR also facilitates practical alignment of medication dosing with work schedules, an especially relevant consideration for patients engaged in shift work, early-morning labor, or cognitively demanding roles. Adjusting dosing times to minimize peak side effects during working hours helps support both adherence and job performance.

Finally, the integration of shared decision-making reinforces treatment engagement. By framing the benefits of therapy in terms that matter to patients—job retention, functional independence, and quality of life—clinicians enhance motivation, strengthen self-management, and promote long-term adherence [4,18]. This collaborative, patient-centered approach ensures that medication optimization is not only clinically appropriate but also occupationally sustainable.

## 10. Legal, Policy, and Employer Engagement

A structured legal and organizational framework is essential to support safe and sustainable return to work (RTW) following a cardiac event. Central to this process is privacy-respecting communication between clinical teams, occupational health services, and employers. When clinicians convey only functional capacities and limitations—without disclosing sensitive medical details—employers can implement reasonable accommodations such as modified tasks, adjusted workloads, reduced or flexible working hours, ergonomic adaptations, and phased duty escalation that aligns with the patient’s evolving physical tolerance [2,3,4]. These measures help balance workplace safety with employee autonomy and are fundamental to compliance with workplace disability and non-discrimination frameworks in many jurisdictions.

For workers with implantable cardioverter-defibrillators (ICDs), specialized policies are often required due to the need to minimize exposure to electromagnetic fields (EMFs) and to ensure that appropriate emergency response plans are in place. This may include restrictions on tasks involving heavy electrical equipment, ensuring safe distances from industrial machinery, and training supervisors in ICD-related emergency protocols. Such tailored policies protect device integrity while allowing individuals to remain engaged in meaningful work roles [2,3,4].

Equally important are social and financial support structures that enable graded, stepwise RTW. Transitional income support, partial-disability benefits, flexible scheduling, and employer-sponsored accommodations reduce financial strain during recovery and enhance adherence to both medical therapy and rehabilitation recommendations [2,3,4]. By reducing economic pressure, these supports help employees avoid premature return or unsustainable work patterns that might jeopardize long-term health.

Collectively, these legal, organizational, and employer-engagement strategies form the backbone of equitable, sustainable RTW pathways, ensuring that clinical recovery is complemented by safe occupational reintegration and preserved quality of life.

## 11. Implementation Toolkit for Clinicians and Employers

A structured and interoperable implementation toolkit is essential to translate cardiac rehabilitation (CR) principles into consistent, high-quality vocational outcomes. Central to this toolkit are standardized intake forms that capture job-specific metabolic demands (job-related MET requirements), shift schedules, environmental exposures, and ergonomic constraints. This information enables clinicians to integrate occupational demands directly with functional assessments. Complementing these intake tools, CPET summary sheets convert measured VO_2_peak values into individualized MET ceilings, allowing precise matching of physical capacity with workplace requirements and supporting safe, evidence-based return-to-work (RTW) planning [4,19].

Systematic tracking tools enhance continuity and quality of care. Work Performance Scale (WPS) monitoring, performed weekly during early reintegration and monthly thereafter, provides real-time insight into work functioning. Symptom action plans—succinct, patient-friendly algorithms for managing chest discomfort, dyspnea, dizziness, or palpitations—empower workers to respond appropriately to physiological changes while reducing unnecessary emergency evaluation. For employers, structured guides for phased reintegration outline recommended increments in hours, task intensity, ergonomic adaptations, and monitoring checkpoints, helping supervisors support RTW safely and consistently.

Innovative approaches such as co-design of CR programs with knowledge users have been advocated to enhance engagement, contextual adaptation, and psychosocial outcomes, especially in underserved populations [52].

Remote and hybrid models benefit from tele-coaching scripts that guide virtual follow-up sessions, ensuring uniform delivery of behavioral counseling, adherence reinforcement, and early problem detection. To support program-level evaluation, integrated dashboards aggregate key indicators including session attendance, VO_2_peak or MET trajectories, WPS trends, and sick leave patterns. These data facilitate early identification of patients at risk of work detachment and enable targeted intervention.

Finally, adherence to accreditation standards ensures that CR programs maintain high fidelity, standardized processes, and outcome transparency—elements shown to drive quality improvement and long-term vocational success [4,19].

The proposed toolkit should be interpreted as a practical framework rather than a prescriptive standard. Adaptation to local healthcare systems, labor regulations, and resource availability is necessary to ensure feasibility and equity across different settings.

## 12. Metrics, Quality, and Accreditation

High-quality CR programs rely on clearly defined, reproducible metrics to evaluate patient progress, program performance, and long-term vocational outcomes. Core performance indicators include changes in VO_2_peak and MET capacity, which serve as objective markers of functional improvement and correlate strongly with both cardiovascular prognosis and work readiness. Parallel tracking of Work Performance Scale (WPS) scores provides insight into real-world occupational functioning, capturing dimensions of reliability, stamina, and cognitive efficiency relevant to sustained return to work (RTW). Additional core measures—such as adherence to CR sessions, timely completion of training modules, and time to RTW—allow clinicians to evaluate the trajectory and durability of recovery [4,19].

Secondary metrics complement these physiological and functional indicators. Routine monitoring with HADS or PHQ instruments identifies early psychological distress that may impede vocational reintegration. Medication persistence serves as a surrogate for treatment engagement and long-term risk reduction, while sick leave days at 3, 6, and 12 months quantify post-event productivity and can highlight emerging barriers to sustained employment. These integrated measures enable a multidimensional understanding of recovery that extends beyond traditional clinical endpoints [4,19].

Accreditation plays a pivotal role in ensuring consistency and accountability across CR programs. Accredited programs adhere to standardized audit processes, data-reporting structures, and minimum quality benchmarks, thereby reducing variability in clinical practice. Evidence shows that participation in short-term, comprehensive CR after acute myocardial infarction is associated with reduced one-year mortality, underscoring the life-saving impact of high-fidelity program delivery [53,54,55].

Together, these metrics and accreditation standards create a framework that supports continuous improvement, promotes equitable access to high-quality care, and strengthens the link between clinical recovery and sustainable workforce participation.

## 13. Limitations and Research Gaps

Despite growing evidence supporting structured return-to-work (RTW) pathways after cardiovascular events, several important limitations remain. First, RTW outcomes vary substantially across countries, largely due to differences in social protection systems, labor market structures, and access to cardiac rehabilitation. Regions with strong employment protections and graded RTW policies tend to show higher long-term retention, whereas areas with limited social safety nets demonstrate higher post-MI job detachment [1]. These contextual differences constrain the generalizability of RTW algorithms and highlight the need for policy-sensitive implementation strategies.

Second, the availability of standardized job-specific MET estimates across industries remains limited. Existing MET tables provide broad approximations but lack granularity for mixed-duty roles, emerging occupations, or jobs with variable physical intensity. This limits the precision of capacity–demand matching and underscores the need for systematic occupational MET catalogues informed by real-world ergonomic and physiological data [4].

A further limitation involves the integration of wearable technologies—such as heart-rate monitors, accelerometers, and recovery-tracking platforms—into clinical decision-making. While these tools could provide continuous, ecologically valid data on exertion and symptom patterns during RTW, their use is not yet standardized, and validation across diverse cardiac populations remains incomplete [5].

Additionally, although psychosocial interventions improve mental health and support RTW, scaling these approaches requires careful attention to treatment fidelity, training standardization, and resource allocation to avoid dilution of therapeutic effect [19].

Finally, there is a major need for longitudinal registries extending beyond 12 months, stratified by occupation, gender, employment type, and socioeconomic factors. Such datasets would enable refinement of prognostic models, strengthen RTW algorithms, and guide evidence-based policy development [1,4,5,19].

## 14. Discussion

Return to work (RTW) after acute coronary syndrome (ACS) or heart failure (HF) is best understood as a clinical–functional–psychosocial trajectory rather than a single binary event. Clinical markers such as left ventricular ejection fraction (LVEF), Killip class, and multimorbidity stratify medical risk, yet they do not fully capture the feasibility or durability of vocational reintegration [8,30]. In contrast, functional capacity—quantified via cardiopulmonary exercise testing (CPET) and referenced to job-specific metabolic equivalents of task (METs)—and psychological readiness jointly determine whether reintegration will be safe and sustained [2,38]. Depression, anxiety, maladaptive illness perceptions, and low return-to-work self-efficacy consistently predict delayed or unsuccessful RTW even in patients with adequate physical performance [7,23,24,25,26,27].

Two pragmatic workload principles operationalize CPET into daily practice: (i) maintaining sustained demands within 35–40% of maximal capacity during 6–8 h shifts and (ii) ensuring peak capacity ≥2× the average job MET requirement to provide sufficient reserve for task peaks [38]. These rules are rooted in fatigue physiology and ensure a metabolic buffer across the workday. Translating them into employer-facing guidance—framed as capacity-based, time-limited, and reassessable—supports safe escalation while preserving confidentiality [2,3].

Evidence from registries, cohorts, and rehabilitation trials consistently positions comprehensive cardiac rehabilitation (CR) as the bridge between clinical recovery and vocational reintegration [2,4,19]. CR improves exercise tolerance, risk factor control, medication adherence, mood, and self-confidence—factors associated with earlier and more stable RTW [14,15,16,17,41]. Registry analyses reveal high nominal RTW within 6–12 months but a notable rate of subsequent detachment, reinforcing sustained RTW—not merely initial return—as the meaningful endpoint [1,2,5]. Telerehabilitation and hybrid models yield functional outcomes comparable to center-based CR while enhancing adherence during the fragile transition back to work [42,43,44].

Divergences in the literature often reflect occupation-specific profiles: blue-collar roles face higher physical demands (handling, lifting, environmental stressors), whereas white-collar roles may concentrate psychosocial burdens (time pressure, low control, effort–reward imbalance) with differential impacts on Work Performance Scale (WPS) trajectories [16,25,41]. Gender disparities persist, with women returning later and less frequently, influenced by clinical, psychosocial, and social role factors [47,48,49]. Younger workers may experience long-term detachment despite early RTW, underscoring the need for tailored support [1].

**Figure 1:**
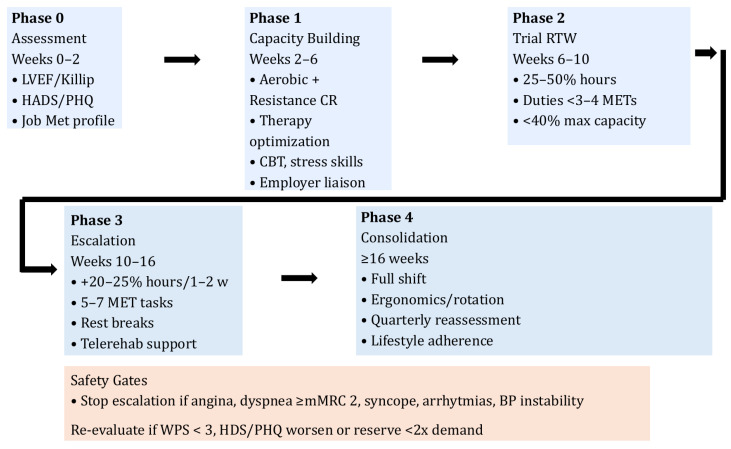
Stepwise return-to-work algorithm within cardiac rehabilitation. Conceptual flow of phased reintegration after ACS/HF with capacity building, psychosocial support, and safety gates. CR = cardiac rehabilitation; CPET = cardiopulmonary exercise testing; MET = metabolic equivalent; WPS = Work Performance Scale [2,3,14,15,16,17,18,34,35,36,37].

CPET-guided workload matching enables clinicians to issue graduated permissions rather than binary fit/unfit decisions. Example prescriptions might specify: Weeks 6–10 at 25–50% hours with tasks < 3–4 METs; Weeks 10–16 add moderate tasks (5–7 METs) with micro-breaks; and escalation is contingent on the absence of angina, dyspnea ≥ mMRC 2, syncope, unstable arrhythmias, or blood pressure instability, and supported by weekly WPS monitoring [14,15,16,17,18,38]. Systematic psychological assessment should be embedded in CR programs from intake, using HADS/PHQ and brief interventions (psychoeducation, behavioral activation, cognitive reframing, graded exposure) to strengthen RTW self-efficacy and reduce fear-avoidance [2,4,19,39,51]. Medication optimization should align dosing with shift patterns, manage side effects relevant to performance (orthostasis, fatigue), and frame adherence benefits in terms of job retention and quality of life [4,18] (Figure 1).

For device carriers (e.g., ICD), counsel must address electromagnetic field exposures, emergency response plans on site, and scheduling of device checks prior to workload escalation, with privacy-respecting communication to employers [2,4,18].

Sustained RTW functions as a population health indicator; it reduces indirect costs, supports social participation, and is correlated with improved long-term outcomes [1,2,22]. Organizations should adopt phased reintegration templates with predictable increments (e.g., +20–25% hours every 1–2 weeks), task rotation, ergonomic adjustments, climate controls, and micro-breaks. Occupational physicians can translate CPET/MET ceilings into duty matrices comprehensible to line managers [2,3,4,19].

Equity considerations—such as gender, socio-economic gradients, blue- vs. white-collar differences—warrant proactive access to CR (flexible scheduling), telerehab options, and supportive policies to maintain adherence without excessive work disruption [42,43,44,45,47,48,49]. Health systems should recognize telerehabilitation as a reimbursable extension of CR, meeting privacy, usability, and interoperability standards [43].

The model integrates risk stratification (LVEF, Killip), CPET-based functional profiling, psychosocial care, telerehabilitation, and employer liaison within a single operational framework [2,8,30,31,32,33,34,35,36,37,38]. This synthesis aligns with exercise physiology and contemporary secondary prevention. Implementation is supported by a toolkit of standardized intake forms (job METs, exposure inventories, shift patterns), CPET summaries (VO_2_peak → MET ceilings), WPS tracking, symptom action plans, and templated employer guidance—facilitating scale-up without loss of fidelity [2,4,19,38,39,40] (Table 2).

**Figure 2:**
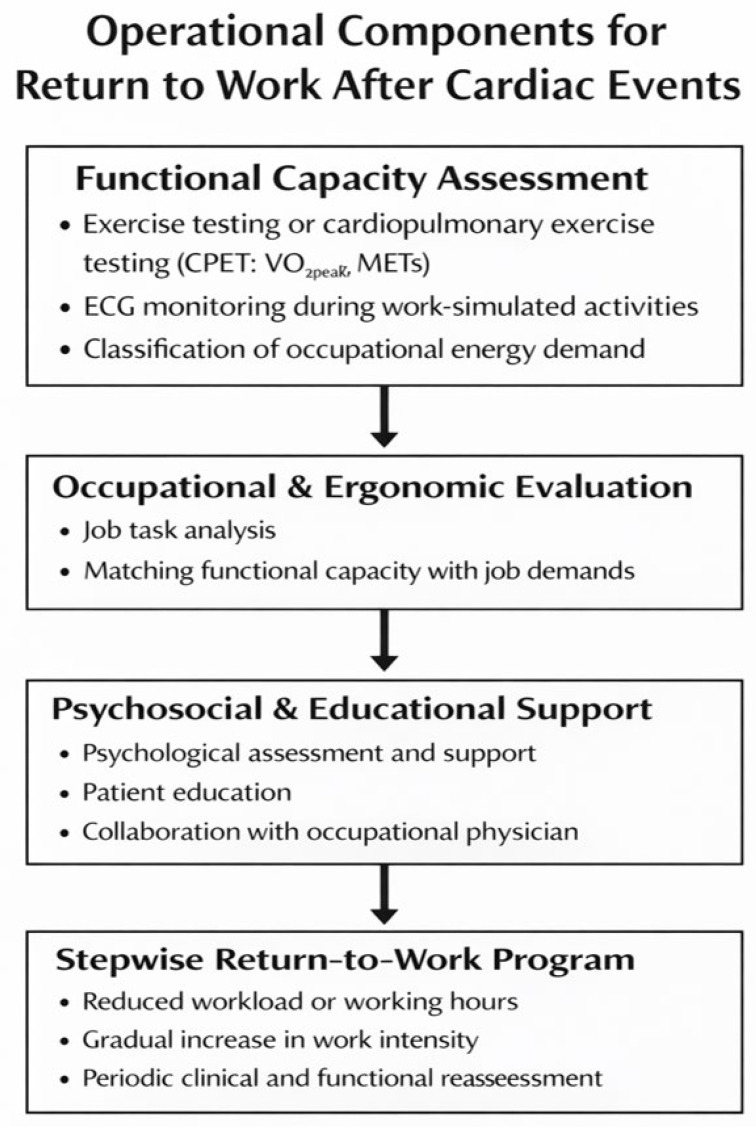
Multidimensional approach to return to work after a cardiac event. The model integrates functional capacity assessment, occupational and ergonomic evaluation, psychosocial support, and a stepwise reintegration strategy to ensure a safe and sustainable return to work.

However, heterogeneity persists across welfare systems, occupations, and CR designs. Non-standardized job-specific MET catalogs limit precision, particularly in mixed-duty or informal roles [38]. Psychosocial and digital interventions vary in dose, content, and delivery, complicating comparisons and pooled inferences [4,19,42,43,44,45]. Many studies restrict RTW assessment to 6–12 months, constraining insight into long-term retention and detachment [1,2,5]. Generalizability beyond high-income contexts remains uncertain and warrants targeted investigation [21,22] (Figure 2).

Interpretation of CR–RTW associations should consider potential methodological biases inherent to observational data. Patients who enroll in or complete CR may differ systematically from non-participants, exhibiting higher baseline motivation, fewer comorbidities, or stronger social support (healthy participant effect). Even when studies adjust for known confounders, residual confounding and unmeasured psychosocial factors may influence observed associations. Clear and consistent definitions of RTW, including thresholds for duration and intensity of work resumption, are essential to enhance comparability and rigor in future research.

Future Directions Priorities include: international standardization of job METs to refine CPET-guided matching [38]; pragmatic trials testing bundled CR–vocational interventions (exercise + CBT + employer liaison) with sustained RTW as primary endpoints [2,4,19,39]; wearable-based monitoring to link real-world workloads with physiological responses for adaptive titration [42,43,44,45]; registries stratified by occupation, gender, and employment type, with ≥24-month follow-up [1,2,5]; and AI-assisted prediction and triage with safeguards for fairness and clinical oversight.

## 15. Conclusions

A multidimensional CR program—integrating capacity-building, MET-matched prescriptions, psychosocial care, telerehabilitation, and employer engagement—offers the most coherent route to safe, stable, and durable RTW after ACS/HF. Centering decisions on reserve and safety gates, and maintaining structured follow-up, aligns individual recovery with organizational needs and societal value [2,18,38,40,53,54,55].

## Figures and Tables

**Table 1:** Workload categories, typical METs, example occupations, and recommended minimum capacity targets.

Workload Category	Typical METs (avg)	Example Occupations	Min VO_2_peak (ml/kg/min) (≈METs × 3.5)	Min Peak METs Target (≥2× avg Demand)
Very light	2.0–2.5	Office clerk, receptionist, call-center	7–9	≥4–5
Light	3.0–4.0	Retail staff, teacher, lab tech, light assembly	11–14	≥6–8
Moderate	5.0–6.0	Nurse on ward, postal carrier, warehouse (mixed)	18–21	≥10–12
Heavy	7.0–8.0	Construction, agriculture, firefighting	25–28	≥14–16

Notes: Average demand should remain at ≤35–40% of maximal capacity for sustained shifts. Targets are indicative and require CPET verification and clinical judgment. References: [2,18,30,32].

**Table 2:** Return-to-work guidance by clinical risk strata (LVEF, Killip class, and comorbidities). Indicative recommendations to align duty intensity with clinical risk and functional reserve; requires CPET verification and individual clinical judgment. References: [8,18,28,29,30].

Risk Stratum	Comorbidity Profile	Suggested RTW Start (Weeks Post-Event)	Duty Intensity (Avg METs)	Min Peak METs Target (≥2× Avg Demand)	Special Conditions/Notes
LVEF ≥ 50%; Killip I–II	Low (no diabetes/CKD; controlled BP)	6–10	≤3–4 (light)	≥6–8	Stepwise hours; monitor WPS weekly; CBT for residual anxiety.
LVEF 40–49%; Killip II	Moderate (one stable comorbidity)	8–12	≤3–4 → 5 (light → moderate)	≥8–10	Rotation of tasks; rest breaks; telerehab check-ins.
LVEF 30–39%; Killip II–III	High (≥2 comorbidities or CKD stage ≥ 3)	10–16	≤3–4 (light) initially	≥10–12	Prolonged supervised CR; defer heavy/manual tasks; safety gates strict.
LVEF < 30%; Killip III–IV	Very high (HF symptoms; frequent exacerbations)	>16 (individualized)	Very light only (≤2.5)	≥5 (if RTW)	Extended rehabilitation; avoid safety-critical roles until stable.
Post–cardiogenic shock (CS) survivors	Variable; assess neurocognitive status	Individualized (often > 16)	Very light → light	≥6–8	Neurocognitive screening; longer observation; gradual cognitive/physical load.
ICD carriers (post-AMI/HF)	Device-dependent risks	Per clinical stability	Role-dependent	Role-dependent	Avoid high-EMF tasks; device checks; emergency plan on site.

Notes: Duty intensity should remain at ≤35–40% of maximal capacity during sustained shifts; peak capacity should be ≥2× average job demand. Use CPET to confirm VO_2_peak/METs and adapt to symptoms, WPS, and hemodynamics. References: [4,18,30,32].

## Data Availability

Data sharing is not applicable.

## Abbreviations

ACS	Acute Coronary Syndrome
AMI/MI	Acute Myocardial Infarction/Myocardial Infarction
CPET	Cardiopulmonary Exercise Testing
CR	Cardiac Rehabilitation
CS	Cardiogenic Shock
GLS	Global Longitudinal Strain
HF	Heart Failure
ICD	Implantable Cardioverter-Defibrillator
LVEF	Left Ventricular Ejection Fraction
MET	Metabolic Equivalent of Task
QoL	Quality of Life
RTW	Return to Work
WPS	Work Performance Scale
